# Common CHD8 Genomic Targets Contrast With Model-Specific Transcriptional Impacts of *CHD8* Haploinsufficiency

**DOI:** 10.3389/fnmol.2018.00481

**Published:** 2019-01-14

**Authors:** A. Ayanna Wade, Kenneth Lim, Rinaldo Catta-Preta, Alex S. Nord

**Affiliations:** ^1^Department of Psychiatry and Behavioral Sciences, University of California, Davis, Davis, CA, United States; ^2^Department of Neurobiology, Physiology and Behavior, University of California, Davis, Davis, CA, United States

**Keywords:** autism spectrum disorder, CHD8, chromatin remodeling, functional genomics, neurodevelopment

## Abstract

The packaging of DNA into chromatin determines the transcriptional potential of cells and is central to eukaryotic gene regulation. Case sequencing studies have revealed mutations to proteins that regulate chromatin state, known as chromatin remodeling factors, with causal roles in neurodevelopmental disorders. Chromodomain helicase DNA binding protein 8 (*CHD8*) encodes a chromatin remodeling factor with among the highest *de novo* loss-of-function mutation rates in patients with autism spectrum disorder (ASD). However, mechanisms associated with *CHD8* pathology have yet to be elucidated. We analyzed published transcriptomic data across *CHD8 in vitro* and *in vivo* knockdown and knockout models and CHD8 binding across published ChIP-seq datasets to identify convergent mechanisms of gene regulation by CHD8. Differentially expressed genes (DEGs) across models varied, but overlap was observed between downregulated genes involved in neuronal development and function, cell cycle, chromatin dynamics, and RNA processing, and between upregulated genes involved in metabolism and immune response. Considering the variability in transcriptional changes and the cells and tissues represented across ChIP-seq analysis, we found a surprisingly consistent set of high-affinity CHD8 genomic interactions. CHD8 was enriched near promoters of genes involved in basic cell functions and gene regulation. Overlap between high-affinity CHD8 targets and DEGs shows that reduced dosage of *CHD8* directly relates to decreased expression of cell cycle, chromatin organization, and RNA processing genes, but only in a subset of studies. This meta-analysis verifies CHD8 as a master regulator of gene expression and reveals a consistent set of high-affinity CHD8 targets across human, mouse, and rat *in vivo* and *in vitro* studies. These conserved regulatory targets include many genes that are also implicated in ASD. Our findings suggest a model where perturbation to dosage-sensitive CHD8 genomic interactions with a highly-conserved set of regulatory targets leads to model-specific downstream transcriptional impacts.

## Introduction

Genetic studies have found that heterozygous loss-of-function mutations to chromatin remodeling genes significantly contribute to autism spectrum disorder (ASD) neurobiology, presumably through disruptions to transcriptional regulation in the developing and mature brain (O’Roak et al., 2012a,b; Parikshak et al., 2013; De Rubeis et al., 2014; Iossifov et al., 2014; Sanders et al., 2015; Vissers et al., 2016). The gene encoding chromodomain helicase DNA binding protein 8 (*CHD8*) has one of the highest observed mutation rates in sporadic ASD (O’Roak et al., 2012a; Krumm et al., 2014; Barnard et al., 2015), and mutations to *CHD8* have also been identified in cases from schizophrenia and intellectual disability cohorts (McCarthy et al., 2014; Tatton-Brown et al., 2017). In addition to primary neurodevelopmental and psychiatric disorder diagnosis, patients that carry *CHD8* mutations present with comorbid macrocephaly, craniofacial dysmorphology, and gastrointestinal pathology (Bernier et al., 2014).

CHD8 belongs to the CHD family of ATP-dependent chromatin remodelers (Hall and Georgel, 2007; Marfella and Imbalzano, 2007; Hargreaves and Crabtree, 2011). CHD family proteins are distinguished by tandem chromodomains predicted to enable histone binding (Flanagan et al., 2005). As some CHD proteins demonstrate chromatin remodeling activity (Tong et al., 1998; Hall and Georgel, 2007; McKnight et al., 2011), CHD8 has been speculated to drive pathological changes in neurodevelopmental gene expression by targeting and remodeling chromatin at specific promoters and enhancers (Sugathan et al., 2014; Ceballos-Chávez et al., 2015; Cotney et al., 2015). This is supported by evidence that CHD8 can reposition nucleosomes *in vitro* and in mammalian cell culture (Thompson et al., 2008).

Several mechanisms have been suggested to underlie CHD8 binding specificity, including through histone modifications associated with open chromatin (Yuan et al., 2007; Rodriguez-Paredes et al., 2009; Sugathan et al., 2014; Cotney et al., 2015) and recruitment via protein–protein interactions (Ishihara et al., 2006; Yuan et al., 2007; Thompson et al., 2008; Nishiyama et al., 2009; Rodriguez-Paredes et al., 2009; Shen et al., 2015; Fang et al., 2016). While the impact of haploinsufficiency on CHD8 function is unclear, loss of *CHD8* in *in vitro* and *in vivo* models dysregulates ASD-associated and CHD8-target gene expression (Sugathan et al., 2014; Cotney et al., 2015; Katayama et al., 2016; Gompers et al., 2017). Whether reported patterns of transcriptional dysregulation associated with *CHD8* haploinsufficiency are due to direct effects versus downstream or secondary changes to CHD8 regulation remains unresolved.

Knockdown or haploinsufficiency of *Chd8* in animal models has recapitulated specific neuroanatomical, gastrointestinal, cognitive, and behavioral phenotypes observed in patients (Sugathan et al., 2014; Katayama et al., 2016; Gompers et al., 2017; Platt et al., 2017), though reported phenotypes vary across models. Published studies encompass *in vitro* and *in vivo* systems and shRNA knockdown or targeted mutation of *CHD8*. Despite the variety of models, there appear to be general patterns of neurodevelopmental disruption caused by reduced *CHD8* expression, characterized by impacts to neuronal proliferation, differentiation, and synaptic function. However, discrepancies between studies make it difficult to reconcile consistent mechanisms and phenotypes.

Characterizing convergent patterns of CHD8 genomic interactions and transcriptional outcomes caused by *CHD8* haploinsufficiency across studies could significantly advance understanding of core pathophysiology associated with *CHD8* mutations and reveal chromatin-associated mechanisms underlying complex brain disorders. While published models of *CHD8* haploinsufficiency vary considerably in design, nearly all have leveraged genomic approaches to determine the impact of reduction of *CHD8* dosage on gene expression. Many have also examined CHD8 interaction targets genome-wide. The methods used, RNA sequencing (RNA-seq) and chromatin immunoprecipitation followed by sequencing (ChIP-seq), generate comparable quantitative data enabling direct comparisons of results across models and studies.

We re-analyzed published RNA- and ChIP-seq data and built an online user interface enabling browsable comparison of gene expression changes linked to *CHD8* haploinsufficiency. Across studies, we found overlapping changes in gene expression across haploinsufficiency models and a strikingly consistent set of high-affinity CHD8 interaction target genes across all binding datasets. The findings of this meta-analysis suggest evolutionarily-conserved and non-cell-type specific high-affinity genomic targets of CHD8 across human, mouse, and rat models. By disrupting these genomic interactions, or by secondary mechanisms, reduction in *CHD8* expression directly and indirectly altered transcription of genes critical for neurodevelopment and previously implicated in neurodevelopmental disorders.

## Materials and Methods

### *CHD8* Genomic Datasets

Next-generation sequencing datasets generated from *CHD8* studies were identified through a literature search with the keyword “CHD8” in PubMed and Gene Expression Omnibus (GEO) databases. Raw data from publications that featured RNA-seq or ChIP-seq analysis were downloaded from GEO with the exception of three publications that hosted raw data on DNA Data Bank of Japan (DDBJ) (Katayama et al., 2016) and Sequence Read Archive (SRA) (Wilkinson et al., 2015; Platt et al., 2017). A total of fifteen publications corresponding to 305 sequencing libraries were included in the analysis. Libraries from Cotney et al. (2015) generated from fetal brain and libraries from Han et al. (2017) designed for analysis of alternative splicing were not included in the analysis. To enable comparison of genomic binding properties, we also included published ChIP-seq data for two brain transcription factors, Nkx2.1 and cFos (Malik et al., 2014; Sandberg et al., 2016). All data included were stated to be in compliance with respective animal care and use committees at time of original publication.

### RNA-seq Analysis

RNA-seq computational analysis was performed following an established pipeline using standard software, as described previously (Gompers et al., 2017). Briefly, unaligned sequencing reads were assessed for general quality using FastQC (Version 0.11.2) and aligned to the mouse (mm9) or human (GRCh37) reference genome using STAR (Version 2.5.2b, Dobin et al., 2013). Aligned reads mapping to genes according to the mm9 genes.gtf or to gencode.v19.annotation.gtf were counted at the gene level using subreads featureCounts (Version 1.5.0-p1, Liao et al., 2014). Overall data quality, including testing for GC-bias, gene body coverage bias, and proportion of reads in exons was further assessed using RSeQC (Version 2.6.4, Wang et al., 2012). Raw gene count data and sample information as reported in the respective repositories were used for differential expression analysis using edgeR (Version 3.4.4, Robinson et al., 2010). Genes with at least 0.1 count per million were included in a general linearized model using a sequencing-run factor-based covariate with genotype or knockdown as the variables for testing. For some datasets, additional covariates were included if described in the original publication. Where possible, overall patterns of differentially expressed genes were compared to the original publication to ensure consistency in results. Normalized expression levels were generated using the edgeR rpkm function. Normalized log_2_(RPKM) values were used for plotting summary heatmaps and for expression data of individual genes. Variation in sequencing depth and intra-study sample variability partially account for differences in sensitivity and power across studies and likely drive some differences observed including the total number of differentially expressed genes (DEGs). To capture an inclusive set of DEGs, DEGs were defined by uncorrected p-values < 0.05. DEG sets were used for gene set enrichment analysis for Gene Ontology via goseq (Young et al., 2010). Enrichment among overlapping DEGs across studies was performed by comparing observed overlap to overlap among randomly selected genes across 1000 permutations.

### ChIP-seq Analysis

ChIP-seq analysis was also performed using an established pipeline and standard methods, as reported before (Gompers et al., 2017). Briefly, unaligned sequencing reads were assessed for general quality using FastQC and mapped to the mouse (mm9), human (hg19), or rat (rn5) genome using BWA (Version 0.7.13, Li and Durbin, 2009). Significant peaks with a *p*-value of <0.00001 were identified using MACS2 (Version 2.1.0, Feng et al., 2011) with model-based peak identification and local significance testing disabled. Test datasets were analyzed comparing each individual ChIP-seq experiment to matched input or IgG controls. Input and IgG libraries were analyzed using the same approach to test for technical artifacts that could confound ChIP-seq results, generally following a previously-reported quality control strategy (Marinov et al., 2014). Enriched regions from IP and control datasets were annotated to genomic features using custom R scripts and the combined UCSC and RefSeq transcript sets for the mouse or human genome build. Regions from the rat genome were lifted over to conserved regions in the mouse genome (mm9). CHD8 target genes were assigned by peak annotation to transcript start site (TSS) or to the nearest TSS for distal peaks. HOMER was used to perform *de novo* motif discovery with default parameters (Version 4.7, Heinz et al., 2010). Where possible, we verified that results from ChIP-seq reanalysis were consistent with original publication.

### Gene Set Enrichment Analysis

For primary analysis, we used the Gene Set Enrichment Analysis tool and the MSigDB database (GSEA, version 3.0, Subramanian et al., 2005) to test for annotated gene sets that show a shift toward tails of log fold change (logFC) rank of RNA-seq or ChIP-seq data. By using this rank-based method, we were able to overcome differences in number of significant genes across datasets. For ChIP-seq, only the top 2000 peaks were used, as enrichment testing is confounded when too large a fraction of included genes are associated. GSEA was used to test for enrichment of gene ontology (GO) and pathway terms. Terms with less than 500 and greater than 20 genes were used, with 1000 permutations tested to determine expected enrichment. Heatmaps showing normalized enrichment score absolute values were plotted for GO and pathway terms for data visualization. As confirmation that top DEGs show similar enrichment to overall rank-based methods, the goseq R package (Version 1.30.0, Young et al., 2010) was used to test enrichment of GO terms for DEGs, correcting for gene length. Analysis required a minimal node size, or number of genes annotated to GO terms, of 20. The internal ‘weight01’ testing framework and Fishers test was used to account for multiple testing comparisons. Test gene sets for DEGs and CHD8 interaction targets were compared against a background set of expressed genes based on the minimum read-count cutoffs for each dataset for DEGs or a background set of all conserved mouse-human genes identified across RNA-seq datasets for CHD8 target genes. Heatmaps showing positive log_2_(observed/expected) values were plotted for GO terms for data visualization. Finally, genes associated with high-affinity CHD8 binding were defined as those present in the top 2000 peaks from any ChIP-seq dataset and were intersected with the Simons Foundation Autism Research Initiative (SFARI) set of ASD risk genes.

### Code and Data Availability and Additional Analysis Visualization

Data that support the findings of this study are available from the corresponding author upon request. Accession numbers in parentheses and DOIs for all published gene sets used in enrichment analysis:

Ceballos-Chávez et al. (2015) (GSE62428): 10.1371/journal.pgen.100517;
Cotney et al. (2015) (GSE57369): 10.1038/ncomms740;
de Dieuleveult et al. (2016) (GSE64825): 10.1038/nature16505;
Durak et al. (2016) (GSE72442): 10.1038/nn.4400;
Gompers et al. (2017) (GSE99331): 10.1038/nn.4592;
Jung et al. (2018) (GSE87370): 10.1038/s41593-018-0208-z;
Katayama et al. (2016) (DRA003116): 10.1038/nature19357;
Platt et al. (2017) (PRJNA379430): 10.1016/j.celrep.2017.03.052;
Shen et al. (2015) (GSE71183, GSE71185): 10.1016/j.molcel.2015.10.033;
Suetterlin et al. (2018) (GSE81103): 10.1093/cercor/bhy058;
Sugathan et al. (2014) (GSE61492): 10.1073/pnas.1405266111;
Wang et al. (2015) (GSE71594): 10.1186/s13229-015-0048-6;
Wang et al. (2017) (GSE85417): 10.1186/s13229-017-0124-1;
Wilkinson et al. (2015) (PRJNA305612): 10.1038/tp.2015.62;
Zhao et al. (2018) (GSE107919): 10.1016/j.devcel.2018.05.022.

Expanded results of the meta-analysis reported here are available from the interactive web server available at https://github.com/NordNeurogenomicsLab/. ChIP-seq datasets available as Track Hubs for upload to the UCSC Genome Browser and analysis scripts are also available at https://github.com/NordNeurogenomicsLab/.

## Results

### Patterns of Transcriptional Pathology Associated With *CHD8* Haploinsufficiency

**FIGURE 1:**
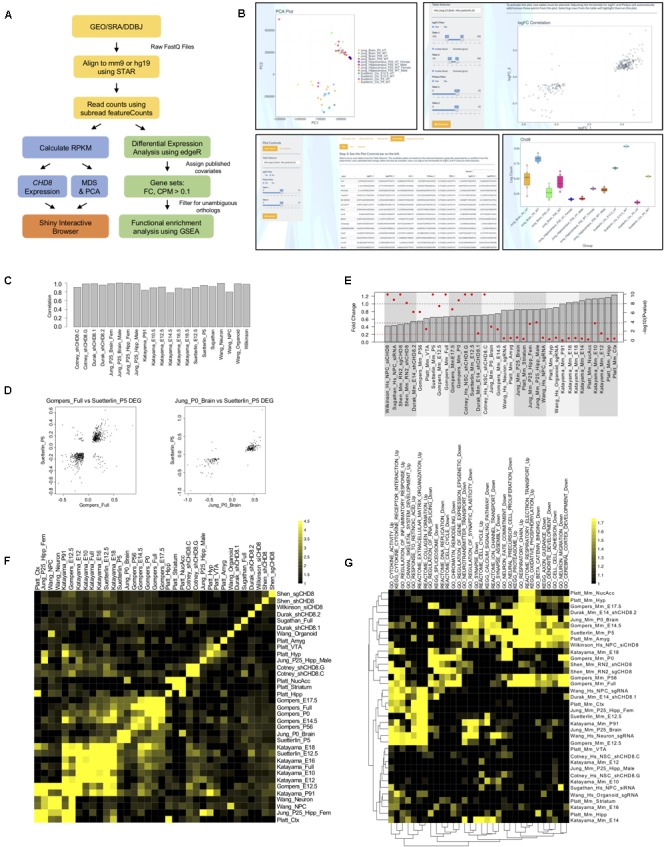
Differential gene expression across *CHD8* models. **(A)** RNA-seq data analysis pipeline. **(B)** Example screen captures of tools available through the R Shiny interactive web browser. Shown are example pairwise comparisons between the Jung et al. (2018) and Suetterlin et al. (2018) RNA-seq datasets. All plots and tables generated using the online interface can be downloaded and analyzed using pseudo counts or relative expression. *Top Left:* Principle component analysis (PCA) showing the first two components separating multiple Jung et al. (2018) and Suetterlin et al. (2018) datasets. Multidimensional scaling (MDS) plots are also available but are not shown. *Bottom Left:* Table showing log fold gene expression changes and significance values for individual genes meeting a *p* < 0.05 cutoff between select Jung et al. (2018) and Suetterlin et al. (2018) datasets. Heatmaps and scatterplots of select gene expression changes are also available but are not shown. *Top Right:* Log fold change scatterplot generated using select Jung et al. (2018) and Suetterlin et al. (2018) datasets for genes meeting a *p*-value < 0.05 criteria. *Bottom Right: Chd8* log fold change bar plot generated for multiple Jung et al. (2018) and Suetterlin et al. (2018) datasets using the interactive web interface. **(C)** Bar plot showing Spearman correlation in fold change between genes identified as significant according to original publication and genes included in current analysis for each RNA-seq dataset. **(D)** Correlation scatterplots between select Gompers et al. (2017) and Suetterlin et al. (2018); Jung et al. (2018) and Suetterlin et al. (2018) datasets. Data are plotted according to log fold change on the *x*- and *y*-axis of genes meeting a *p* < 0.05 statistical cutoff. **(E)** Change in *CHD8* mRNA across models. Data plotted according to fold change, as indicated by the gray bars, with gray dotted lines indicating 0.5- and 1-fold change. Data are also plotted according to -log(10) *p*-value, as indicated by red dots for each gray bar, with a red dotted line indicating a significance value of *p* < 0.05. Red dots above the red dotted line represent *CHD8* fold changes meeting a *p* < 0.05 cutoff. Hs, human; Mm, mouse. **(F)** Heatmap showing enrichment of genes meeting a *p* < 0.05 statistical threshold between included RNA-seq datasets. The legend indicates log_2_(observed/expected) enrichment. **(G)** Heatmap showing enrichment of gene ontology and pathway terms across RNA-seq datasets using GSEA. Included datasets are plotted on the *y*-axis. Significant terms are plotted on the *x*-axis for downregulated gene sets and upregulated gene sets separately, as indicated with “_Up” and “_Down” suffixes, respectively. The legend indicates absolute value normalized enrichment scores. Data are hierarchically clustered according to similarity as indicated by the dendrograms. Hs, human; Mm, mouse.

We reanalyzed a total of 254 RNA sequencing libraries corresponding to 12 studies of *CHD8* knockdown or heterozygous mutation (Table 1). Almost all datasets represented neuronal model systems except for one dataset using an acute myeloid leukemia cell line (Shen et al., 2015). Analysis of all datasets was performed using the same pipeline with quality control steps and study-specific exceptions for consistency as well as covariate and batch structure as described in original publication (Figure 1A). Results for differential expression testing across all genes and studies included in this analysis are available via our interactive web site (Figure 1B) and included as Supplementary Table S1.

**Table 1:** Summary of RNA-seq datasets included in the *CHD8* model reanalysis.

	Manipulation	Study	Model	System	Timepoint(s)	Notable phenotypes
*CHD8* Knockdown	–	Cotney et al., 2015	H9-derived hNSCs	shRNA transfection	–	–
	–	Durak et al., 2016	Swiss Webster mice: GFP+ Cortical Cells	E13 shRNA electroporation	E15	Decreased Proliferation, Social Impairment, Reduced Exploration, Reduced Dendrite Arborization
	–	Sugathan et al., 2014	iPSC-derived hNPCs	shRNA transfection	–	Macrocephaly, Increased Proliferation (Performed in Zebrafish)
	–	Wilkinson et al., 2015	SK-N-SH hNeuroblastoma cell	siRNA transfection	–	–
	–	Shen et al., 2015	MLL-AF9/NrasG12D mAML (RN2) cells	shRNA transfection	–	–
Heterozygous *CHD8* Mutation	5bp-deletion in *Chd8* Exon 5	Gompers et al., 2017	C57BL/6N mice: Bulk Forebrain	CRISPR-cas9	E: 12.5, 14.5, 17.5 P: 0, 56	Megalencephaly, Increased Proliferation, Cognitive Impairment, Altered RNA Processing
	Deletion in *Chd8* Intron 36/37	Jung et al., 2018	C57BL/6J mice: Whole Brain, Hipp	Cre-LoxP Recomb. (ESC clones injected into blastocysts)	P0, P25	Sex-Dependent Effects, Altered Synaptic Function, ASD-Relevant Maternal Effects
	*Chd8* Exon 11–13 Deletion	Katayama et al., 2016	C57BL/6J mice: Whole Brain	Cre-LoxP Recomb. (ESC clones injected into blastocysts)	E: 10, 12, 14, 16, 18P: 91	Megalencephaly, Increased Anxiety, Persistence, Social Impairment
	7bp-deletion in *Chd8* Exon 1	Platt et al., 2017	C57BL/6J mice: Ctx, Striat, Nuc Acc, VTA, Hipp, Amyg, Hyp	CRISPR-cas9	P70–84	Craniofacial Abnormalities, Megalencephaly, Increased Anxiety, Increased Motor Learning
	*Chd8* Exon 12 or 17 Deletion	Shen et al., 2015	MLL-AF9/NrasG12D mAML (RN2) cells	CRISPR-cas9	–	–
	*Chd8* Exon 3 Deletion	Suetterlin et al., 2018	C57BL/6J mice: Ctx	Cre-LoxP Recomb. (ESC clones injected into blastocysts)	E12.5, P5	Craniofacial Abnormalities, Megalencephaly, Growth Delay, Abnormal Activity Levels, Increased Brain Connectivity
	2 bp- or 10 bp-deletion in *Chd8* Exon 1	Wang et al., 2015	iPSC-derived hNPCs, hNeurons	CRISPR-cas9	–	–
	2 bp- or 10 bp-deletion in *Chd8* Exon 1	Wang et al., 2017	iPSC-derived hCerebral Organoids	CRISPR-cas9	–	–

*h, human; NSCs, neural stem cells; iPSC, induced pluripotent stem cell; NPC, neural progenitor cell; E, embryonic day; P, postnatal day; Ctx, prefrontal cortex; Striat, dorsal striatum; Nuc Acc, nucleus accumbens; VTA, ventral tegmental area; Hipp, hippocampus; Amyg, amygdala; Hyp, lateral hypothalamus; bp, base pair; recomb., recombination; aml, acute myeloid leukemia*.

We verified that differential expression generated in our re-analysis here mirrored original publications using Spearman correlation for logFC of DEGs between the original and re-analysis, shown in Figure 1C. Unsurprisingly, relative gene expression levels varied widely across studies, with principle components of variation dominated by species of origin and experiment (data not shown). Pairwise comparisons between DEGs from individual datasets revealed specific similarities in gene expression changes. For example, comparison of DEGs at the *p* < 0.05 cutoff level between Gompers et al. (2017); Jung et al. (2018), and Suetterlin et al. (2018) datasets revealed strong positive correlations in the direction of differential gene expression, where genes that were significantly up- or down-regulated in one dataset followed the same pattern in the other (Figure 1D). We tested the similarity between DEGs across studies, finding significant overlap across some studies, with the strongest overlap among datasets testing the impact of heterozygous *Chd8* mutation on mouse embryonic and postnatal brain (Figure 1F).

Further pairwise comparisons between studies and expression for specific genes can be done using our interactive web browser available at https://github.com/NordNeurogenomicsLab/. This interactive resource allows for analysis of principle components, differential expression of individual genes, and overall differential expression patterns for all included datasets (Figure 1B).

Considering expression of *CHD8* itself, most knockdown and heterozygous knockout models resulted in a 20% or greater significant decrease in mRNA (Figure 1E). However, published data from some models showed a subtler decrease or even a significant increase in *CHD8*. As stated before, we verified that these findings were consistent with originally-published RNA-seq data. Protein-level validation of *CHD8* dosage decrease was performed in all original publications to confirm *CHD8* haploinsufficiency in each model, but the results are difficult to compare considering the use of different and unvalidated CHD8 antibodies across studies. The absence of reduced *CHD8* mRNA expression for some studies raises questions regarding what expectations should be for gene dosage models.

Across all studies, upregulated and downregulated DEGs passed inclusive (*p* < 0.05), moderate (FDR < 0.1), and stringent (FDR < 0.05) thresholds, though numbers of DEGs varied widely (Supplementary Figure S1A). Large differences in number and effect size of DEGs across studies may be a result of differences in experimental design, impact of knockdown and knockout on *CHD8* dosage, methods, and statistical sensitivity related to intra-study sample variability and sequencing depth. Variability in gene expression could also be due to differences in sensitivity to *CHD8* dosage between developmental stages and type of model used to carry out these experiments.

### Enriched Functional Gene Sets Associated With *CHD8* Haploinsufficiency

We next performed gene set enrichment analysis of biological pathways and Gene Ontology (GO) terms using GSEA and goseq (Figure 1G, Supplementary Tables S2, S3 and Supplementary Figures S1B–D). While relatively small numbers of individual genes showed overlapping significant changes in expression across pairwise study comparisons, we found strong correlation in DEG functional groups across studies. This analysis identified four general signatures across published models. The majority of datasets exhibited one or more of these signatures. Upregulated signatures included immune response and energy metabolism. Downregulated signatures included cell cycle, chromatin organization, RNA processing, neuronal differentiation, and synaptic signaling. These patterns were also present when comparing only the DEGs using goseq rather than GSEA, which uses logFC rank. The general pattern of enriched functional groups held across lenient (Supplementary Figures S1B,C) and stringent (Supplementary Figure S1D) statistical thresholds (Supplementary Tables S2, S3). Clustering of datasets by enrichment for representative terms and biological pathways according to GSEA are shown in Figure 1G.

Of 36 total datasets, around 11 had synaptic or neurodevelopmental terms enriched, 7 had cell cycle, chromatin organization, and RNA processing terms enriched, 11 had a combination of both, and 7 had neither trend represented when analyzed using GSEA (Figure 1G). In addition, 13 had strong enrichment of upregulated pathways, but these datasets tended not to have enrichment of neuronal or gene regulatory pathways. The trend of enrichment of these signatures in the GSEA and goseq datasets showed some correlation to the model system used in each study. *In vitro* models were more likely to have neuronal terms or fail to have a trend represented while *in vivo* models were more likely to have both, or only gene regulation associated terms, represented. There is also some indication that *in vivo* models of postnatal brain tended to have more enrichment of neuronal terms while models of embryonic brain were more likely to also have enrichment of terms associated with gene regulation. Overall, our results suggest that *CHD8* knockdown or heterozygous knockout produces model-specific differential gene expression, with overlap present among general functional classes. Expression changes appear to be more consistent in embryonic and postnatal brain tissues of germline *Chd8* haploinsufficient mice across studies.

### CHD8-DNA Interactions Occur Throughout the Genome Enriched for Promoters

We reanalyzed a total of 51 ChIP-seq sequencing libraries from nine studies of CHD8 genomic interaction patterns (Table 2). Analyzed datasets represented both neuronal and non-neuronal model systems. We included both *in vivo* tissue preparations and *in vitro* culture models from neuronal and non-neuronal cell fates to allow additional examination of tissue or cell-type specificity of CHD8 interactions. Five of the datasets were generated from bulk mouse tissue at adult (3 studies; Katayama et al., 2016; Gompers et al., 2017; Platt et al., 2017) and embryonic (2 studies; Cotney et al., 2015; Katayama et al., 2016) timepoints allowing for investigation of CHD8 interactions *in vivo* across time. Other data were generated from cellular models, with 2 studies using human neuronal lineage cells (Sugathan et al., 2014; Cotney et al., 2015), 2 using mouse or human cancer cell lines (Ceballos-Chávez et al., 2015; Shen et al., 2015), and 1 using mouse embryonic stem cells (de Dieuleveult et al., 2016). Finally, we included 1 cell-type-specific ChIP-seq study profiling oligodendrocyte precursors and mature oligodendroctyes isolated from rat brain (Zhao et al., 2018). As control comparisons, we also included ChIP-seq datasets for neurodevelopmental (Nkx2.1) and activity-dependent neuronal (cFos) transcription factors (Malik et al., 2014; Sandberg et al., 2016).

**Table 2:** Summary of CHD8 datasets included in ChIP-seq reanalysis.

Fragmentation method	Study	Model	Timepoint(s)	Tissue collected	Antibody	Control
Sonication	Cotney et al., 2015	C57BL/6J mice; H9-derived hNSCs	E17.5; –	Frontal Cortex; –	αCHD8 (Abcam, ab114126)	Input
Sonication and Mnase	Katayama et al., 2016	C57BL/6J mice (*Chd8*^+/-^ and WT)	E14, P91	Whole Brain	αCHD8 (Custom)	Input
Sonication	Platt et al., 2017	C57BL/6J mice (*Chd8*^+/-^ and WT)	P70-77	Somatosensory Cortex	αCHD8 (Novus Biologicals, NB100-60417)	IgG
Sonication	Ceballos-Chávez et al., 2015	hT47D-MTVL breast cancer cell	Before progestin stimulation	–	αCHD8 (Bethyl, A301-224A)	IgG
MNase	de Dieuleveult et al., 2016	mESCs with FLAG/HA-tagged CHD8	–	–	αFLAG and αHA	Input
Sonication	Gompers et al., 2017	C57BL/6N mice	∼P56	Bulk Forebrain	αCHD8 (Abcam, ab114126)	Input
Sonication	Shen et al., 2015	mRN2 cells	–	–	αCHD8 (Bethyl, A301-224A)	Input
Sonication	Sugathan et al., 2014	iPSC-derived hNPCs	–	–	αCHD8 (Bethyl, A301-224A; Novus Biologicals, NB100-60417, NB100-60418)	Input
Sonication	Zhao et al., 2018	Rat Cell Culture	–	Cortex	αCHD8 (Abcam, ab114126)	IgG

*All datasets were performed using formaldehyde or another similar method of crosslinking before fragmentation and immunoprecipitation; E, embryonic day; P, postnatal day; h, human; NSCs, neural stem cells; m, mouse; ESCs, embryonic stem cells; HA, haemagglutinin; MNase, micrococcal nuclease; Chd8^+/-^, CHD8 heterozygous mutation carrier.*

**FIGURE 2:**
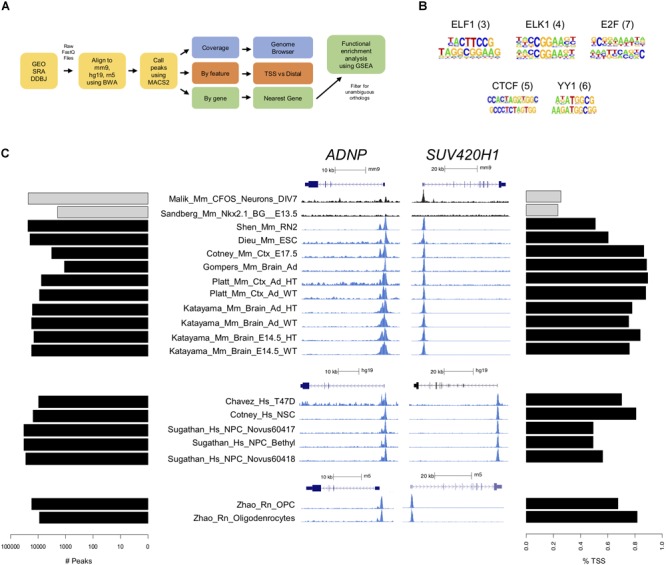
CHD8 binds to promoters across the genome. **(A)** ChIP-seq analysis pipeline. **(B)** Motifs identified in CHD8-bound regions. ELF1, ELK1, E2F, CTCF, and YY1 transcription factors were most commonly represented across datasets. The numbers in parentheses indicate the number of datasets with that motif identified. **(C)** Plots showing the number of peaks with CHD8 binding and preferential promoter binding by CHD8. Each row corresponds to one dataset. Each dataset is identified by name toward the middle of the panel. *Left:* Horizontal bar plot showing the number of significant peaks (MACS2 cutoff of *p* < 0.00001) identified. Control cFos (Malik) and Nkx2.1 (Sandberg) datasets are indicated with gray bars. *Middle:* CHD8 binding near promoters of select chromatin remodeling genes (*ADNP, SUV420H1*) in the mouse, human, and rat ChIP-seq datasets. Two control dataset tracks indicated in black show cFos (Malik) or Nkx2.1 (Sandberg) binding. Linear representations of each gene for each respective genome is indicated above each browser capture grouping and under each respective scale bar. Height of the *y*-axis is scaled to show the peak for each track. *SUV420H1* is *Kmt5b* in rat. *Right:* Horizontal bar plot showing percentage of significant peaks overlapping with the transcription start site of the nearest gene. Control cFos (Malik) and Nkx2.1 (Sandberg) datasets are indicated with gray bars.

ChIP-seq data were analyzed using the same steps for immunoprecipitated, or experimental, and control data in our analysis pipeline (Figure 2A). *De novo* motif analysis performed on CHD8 peak regions across experiments identified various general promoter-associated transcription factor binding sequences, but no clear primary binding motif for CHD8 (Figure 2B). These findings are consistent with original publications, none of which identified a strong candidate primary binding motif, suggesting that CHD8 interactions are not mediated by direct DNA sequence recognition.

There was large variation in number of called peaks, likely due to experimental design and technical differences (Figure 2C, Left). Eleven of the control ChIP-seq libraries were found to have more than 250 called peaks with strong promoter enrichment (Supplementary Figure S2), suggesting some level of technical artifact associated with chromatin preparation (Marinov et al., 2014). Of note, experiments with the largest number of peak calls in the control datasets were among the CHD8 ChIP datasets with the largest number of peaks. Across all ChIP-seq datasets, CHD8 genomic interactions most commonly occurred near promoters (Figure 2C, Right and Supplementary Figure S2). Furthermore, binding to promoter-defined peaks tended to approach 100% as the number of called peaks decreased, suggesting that higher-affinity interactions for CHD8 are strongly biased to promoters. In comparison, the 2 control transcription factor datasets showed much higher proportion of non-transcription start site (TSS) binding. Consistent with original studies that compared Chd8 binding in WT and heterozygous *Chd8* mutant mouse brain, no difference between genotypes was identified, suggesting that haploinsufficiency doesn’t have a strong impact on Chd8 genome-wide interaction patterns. Increased affinity and frequency of promoter interactions by CHD8 was clearly evident in the coverage data signal for mouse tissues, human cell lines, and rat cell culture (Figure 2C, Middle), with *ADNP* and *SUV420H1* loci shown as examples.

### Strong Correlation of CHD8 Genomic Interaction Across Studies Suggests Conserved Regulatory Targets

**FIGURE 3:**
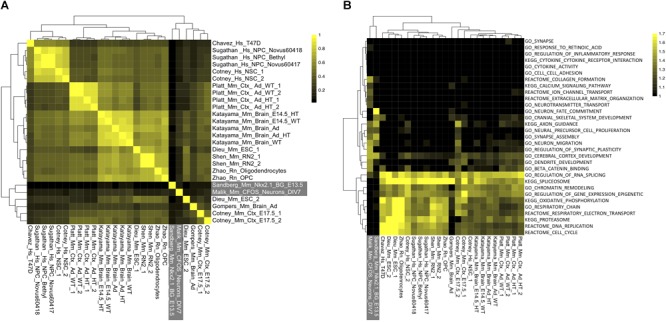
Unexplained specificity of CHD8 binding near chromatin, RNA processing, cell cycle, and metabolism promoters across CHD8 ChIP-seq datasets. **(A)** Heatmap showing correlation across included CHD8 and control ChIP-seq datasets. Legend indicates the correlation between datasets. **(B)** Heatmap showing enrichment of gene ontology and pathway terms for the top 2000 significant peaks meeting a MACS2 significance cutoff of *p* < 0.00001 across ChIP-seq datasets using GSEA. Legend indicates absolute value normalized enrichment scores. Data are hierarchically clustered as indicated by the dendrograms. Control datasets for both panels are indicated in white font outlined by a gray box.

In contrast to variable differential expression changes across RNA-seq studies, CHD8 binding targets were strikingly consistent, particularly among high-affinity peaks (as defined by peak rank). Overall correlation (Spearman’s coefficient) was compared for target genes (defined via TSS peak or as the nearest TSS to a distal peak, Figure 3A). There was correlation across all CHD8 ChIP-seq datasets, with reduced correlation for datasets with fewer peaks and the highest correlations for datasets with the largest number of peaks. In comparison, Nkx2.1 and cFOS showed little correlation to each other or any of the CHD8 datasets. At the level of individual genes, high-affinity targets showed remarkable consistency across all datasets (Supplementary Table S4).

Some of the datasets showed ubiquitous binding across the genome and others showed much smaller target sets, consistent with differences described in original publications. While the number of interactions varied, specific targets and their rank were largely the same. In other words, the strongest interactions were conserved across all CHD8 ChIP-seq datasets. Focusing on high-affinity, or top-ranked targets, we tested these datasets for functional enrichment. GSEA analysis of the top 2000 peaks showed that these high-affinity regulatory interaction targets were overwhelmingly genes involved in RNA and protein processing, cell cycle, chromatin organization, transcription, and metabolism (Figure 3B). In contrast, these genes did not show enrichment in the targets of Nkx2.1 or cFos, indicating this pattern is specific to CHD8.

Comparing the functional terms enriched in the differential RNA across studies, CHD8 appears to directly target and activate genes associated with most of these basic cellular processes. The CHD8 datasets included a number of cell-type specific experiments, for example the oligodendrocyte and oligodendrocyte precursor experiments. We did not observe a difference in the high-affinity targets in these datasets. This suggests that CHD8 shows high-affinity for a remarkably conserved set of promoters from embryonic stem cells to differentiated oligodendrocytes.

### Relationship Between Genomic Interaction and Gene Expression Changes

**FIGURE 4:**
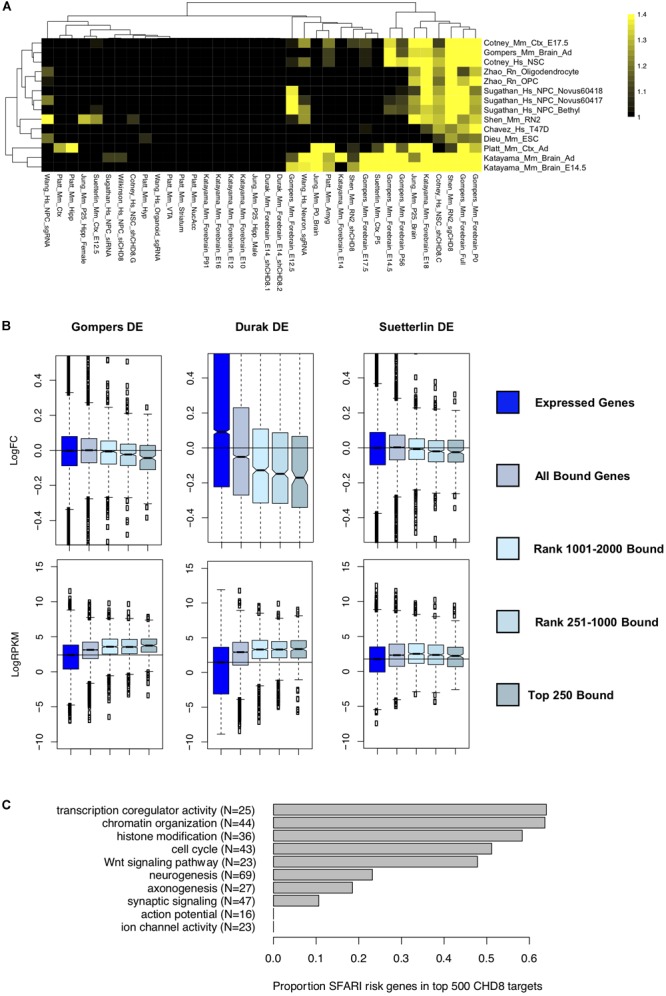
Comparing differentially expressed genes in *CHD8* models to high-affinity CHD8 interactions. **(A)** Heatmap showing correlation between the top 500 rank-ordered significant CHD8 peaks for each ChIP-seq dataset with genes meeting a *p* < 0.05 significance cutoff in each RNA-seq dataset. The legend indicates absolute value normalized enrichment score. Enrichment is comparable using the top 2000 genes. Data were hierarchically clustered according to dataset similarity. **(B)** Comparison between the Platt et al. (2017) Chd8 ChIP-seq dataset and the Gompers et al., 2017 (*Left*), Durak et al. (2016) (*Middle*), and Suetterlin et al. (2018) (*Right*) differential expression gene sets. *Top:* Change in log fold change expression of genes according to CHD8 binding. *Bottom:* Change in log_2_RPKM sequence coverage of genes according to CHD8 binding. Boxes were plotted according to CHD8 binding affinity bins: all genes meeting at least 0.1 count per million sequencing coverage (Expressed Genes), any genes having CHD8 binding (All Bound Genes), and all genes having binding ranked according to CHD8 peak significance (Top 250 Bound, Rank 251–1000 Bound, Rank 1001–2000 Bound). **(C)** Bar plot showing the total number of SFARI risk genes, in parentheses, annotated to select gene ontology terms, and the proportion of SFARI genes bound by CHD8 for each ontology terms, as indicated by the gray bars.

Most genes with CHD8 interactions at or distal to the promoter did not exhibit significant changes in gene expression, regardless of the study, suggesting that there are additional determinants of target gene sensitivity to *CHD8* dosage (Figure 4A). However, we did observe specific overlap between regulatory targets and downregulated genes among a subset of the studies (Figure 4B, top and Supplementary Table S5). Upregulated DEGs, including those involved in metabolism, were not enriched for CHD8 genomic interactions.

For datasets exhibiting downregulation of target genes, there was a clear relationship between strength of CHD8 binding, increased gene expression, and downregulation with *CHD8* haploinsufficiency. Fifteen out of the 36 analyzed datasets showed this trend (Supplementary Figure S3). For instance, an increased signature of downregulation was observed as CHD8 target affinity increased with three studies of *in vivo Chd8* knockdown or heterozygous knockout in mouse brain (Durak et al., 2016; Gompers et al., 2017; Suetterlin et al., 2018; Figure 4B). Regardless of the experiment, CHD8 interaction affinity was also strongest for genes that were more highly expressed (Figure 4B, bottom and Supplementary Figure S4). However, high levels of expression alone did not predict CHD8 interaction or DEG, indicating that expression level does not solely determine CHD8 interactions or sensitivity of regulatory targets to reduced *CHD8* dosage.

While individual studies of the role of *CHD8* haploinsufficiency in neurodevelopmental disorders all highlight strong enrichment of ASD-relevant genes among DEGs, our data suggests that this signal can be separated into direct genomic interaction targets and more brain- and neuron-specific genes. We tested this by looking at the overlap of high-affinity CHD8 targets with genes annotated as high-confidence ASD genes in the SFARI gene database (Figure 4C). Indeed, we found a large proportion of ASD-relevant genes involved in chromatin organization and cell cycle among high-affinity CHD8 targets. In comparison, SFARI ASD genes associated with neurogenesis, axonogenesis, and synaptic signaling showed much lower representation in the high-affinity target sets.

## Discussion

This meta-analysis of published genomic datasets from *in vitro* and *in vivo* mouse, human, and rat studies revealed both consistent and study-specific effects of *CHD8* haploinsufficiency on gene expression and largely concordant high-affinity CHD8 genomic interaction loci. Our results illustrate both the power and limitation of comparing genomic datasets and challenge previous assumptions regarding the regulatory mechanisms and transcriptional pathology associated with *CHD8* haploinsufficiency.

Knockdown or heterozygous mutation of *CHD8* led to characteristic changes in gene expression across studies and model systems. At the gene-by-gene level, these expression changes varied considerably between *CHD8* models, especially when considering lenient (*p* < 0.05) vs. stringent (FDR < 0.05) statistical thresholds. Future work should take this into consideration when analyzing differential gene expression data from *CHD8* models. Compared to the high variability across *in vitro* models, the impact of germline heterozygous *Chd8* mutation in mouse brain was much more consistent, with four of the five studies showing significant DEG overlap. At the level of gene set enrichment, we found global patterns of transcriptional dysregulation with downregulation of genes involved in gene regulation and neuronal development and function and upregulation of genes involved in immune signaling and metabolism.

In contrast to differences in the RNA data, the ChIP-seq results were highly consistent for high-affinity genomic interactions. Comparison across ChIP-seq experiments shows that CHD8 preferentially targets promoters, with no evidence of direct binding through a specific DNA motif. Peaks with the highest signal were constant across experiments, regardless of the model, suggesting that CHD8 preferentially interacts with promoters of a set of genes linked to cellular processes such as those involved in cell cycle, chromatin organization, and RNA processing. We found reduced transcription of these CHD8 target genes in some models, though our data also highlight widespread genomic promoter interactions for CHD8 without obviously strong transcriptional impact from *CHD8* haploinsufficiency for most targets.

While the clear concordance in high-affinity genomic CHD8 interactions suggests common regulatory functions across cell types, it remains to be examined whether the observed dysregulation of neurodevelopmental disorder-relevant neuronal genes is related to context-dependent CHD8 regulatory activity in the brain given the current cellular heterogeneity and technical challenges existing with available CHD8 ChIP-seq. Of note, we did not see enrichment among high-affinity or low-affinity targets for cell-type-specific genes in the datasets examined here, which include some cell-specific analyses. Possible explanations for changes in expression for genes that are not high-affinity CHD8 targets include secondary impacts, increased dosage sensitivity for lower-affinity genomic interaction targets, or a function of CHD8 that is not dependent on specific genomic interactions.

We note a number of technical issues that impacted this meta-analysis, many of which are associated with variation in methods and sequencing depth. Surprisingly, we found considerable differences in *CHD8* expression across models despite the use of common design strategies for testing the impacts of haploinsufficiency. Though we did not find an obvious correlation between *CHD8* transcript levels and up- or downregulated gene expression, it seems likely that differences in experimental design, including *CHD8* knockdown or knockout, contributed toward meaningful variation between models. We also noted differences in ChIP-seq datasets that suggest very different genome-wide binding patterns depending on the experiment. We note that enrichment in control libraries was present across several published datasets, which could confound CHD8-specific peak discovery. Different studies also used various CHD8 antibodies with unknown and unvalidated CHD8 specificities. Nonetheless, by examining patterns across datasets, we identified consistent patterns of enrichment suggesting that overall findings from ChIP-seq targeting CHD8 reliably identify common high-affinity interactions.

It is clear from previous publications and this meta-analysis that CHD8 is critical for neurodevelopment. However, despite the limitations of comparing genomic datasets across variable models, our analysis challenges the simple model that cell-specific CHD8 genomic interaction patterns drive differences in the impact of *CHD8* haploinsufficiency. Our results suggest that, as a chromatin remodeler, CHD8 primarily targets genes involved in cell cycle, chromatin organization, and RNA processing regardless of cell type. Therefore, as an essential gene with widespread expression across neurons and glia, homozygous loss of *CHD8* would likely impact cellular viability in general while heterozygous mutation or knockdown would have subtler, more unpredictable, impacts depending on the cellular context. Such a model would explain the widespread changes in gene expression across model systems and varied reports of impact on proliferation depending on dosage. Nonetheless, given the limitations of current studies we cannot rule out the possibility of cell-type or context-dependent specificity of CHD8 function.

Our results raise two questions that could be addressed by application of RNA-seq and ChIP-seq in the future: (1) What are the developmental stage, cell-type, and region-specific impacts of *CHD8* haploinsufficiency in the developing and mature brain, and (2) Does CHD8 have context-dependent function in specific stages, cell types, and regions with regard to genomic interaction patterns? Beyond addressing these two key issues, additional clarity regarding the role of CHD8 in the brain will come from studies examining molecular and biochemical properties underlying CHD8 function in the brain. As *CHD8* haploinsufficiency may represent common features of haploinsufficiency of other general chromatin remodelers implicated in ASD, further characterization of *CHD8* models and CHD8 genomic interactions could reveal essential functions driving pathology in neurodevelopmental disorders.

## Author Contributions

AW and AN conceived of the project. AW, KL, and AN performed analysis of RNA-seq experiments. AW, RC-P, and AN performed analysis of ChIP-seq experiments. AW and AN drafted the manuscript. All authors contributed to manuscript revision.

## Conflict of Interest Statement

The authors declare that the research was conducted in the absence of any commercial or financial relationships that could be construed as a potential conflict of interest.

## Supplementary Material

FIGURE S1 Consistency in RNA-seq results with goseq analysis across different statistical thresholds. **(A)** Bar plots showing log-scaled differentially expressed gene numbers for uncorrected *p*-value < 0.05 (*top*), FDR < 0.1 (*middle*), and FDR < 0.05 (*bottom*) thresholds with downregulated gene counts in black bars and upregulated gene counts in gray bars. **(B–D)** Heatmaps showing enrichment of gene regulation, neuronal function, and neurodevelopmental gene ontology terms when analyzing genes meeting a significance cutoff of *p* < 0.05 **(B)**, FDR < 0.1 **(C)**, and FDR < 0.05 **(D)** using the goseq statistical package. Included datasets are plotted on the *x*-axis. Significant terms are plotted on the *y*-axis for downregulated gene sets and upregulated gene sets separately, as indicated with “Up” and “Down,” at the beginning of each listed ontology term. The legend indicates log_2_(observed/expected) enrichment. Data were hierarchically clustered according to dataset similarity and term enrichment as indicated by dendrograms on the *x*- and *y*-axis. Ontology terms were selected to match terms for gene set enrichment analysis in Figure 1. Hs, human; Mm, mouse.

FIGURE S2 Significant peak number and preferential promoter binding by CHD8 (or control proteins) for all ChIP-seq datasets. Horizontal bar plots show the number of peaks meeting a MACS2 significance cutoff of *p* < 0.00001 **(Left)** and percentage of significant called peaks overlapping with the transcription start site of the nearest gene **(Right)**. Control cFos (Malik) and Nkx2.1 (Sandberg) datasets are indicated with gray bars.

FIGURE S3 Remaining log fold change plots from the CHD8 binding by differential gene expression comparison analysis. All datasets were analyzed using the Platt et al. (2017) Chd8 ChIP-seq dataset. Datasets shown are from the *CHD8* RNA-seq analysis and are loosely organized based on overlap between downregulated genes, no clear trend, or upregulated genes from top to bottom, which sometimes spanned multiple rows. Each plot shows log fold change on the *y-*axis and CHD8 binding affinity bin on the *x*-axis for each dataset as indicated by name above each plot. CHD8 binding affinity bins: all genes meeting at least 0.1 count per million sequencing coverage (Expressed Genes), any genes having CHD8 binding (All Bound Genes), and all genes having binding ranked according to CHD8 peak significance (Top 250 Bound, Rank 251–1000 Bound, Rank 1001–2000 Bound). Full models for certain datasets were chosen as they exhibited similar signal as the individual timepoint or brain region datasets.

FIGURE S4 Remaining RPKM plots from the CHD8 binding by differential gene expression comparison analysis. All datasets were analyzed using the Platt et al. (2017) Chd8 ChIP-seq dataset. Datasets shown are from the *CHD8* RNA-seq analysis. Each plot shows normalized log_2_RPKM on the *y*-axis and CHD8 binding affinity bin on the *x*-axis for each dataset as indicated by name above each plot. CHD8 binding affinity bins: all genes meeting at least 0.1 count per million sequencing coverage (Expressed Genes), any genes having CHD8 binding (All Bound Genes), and all genes having binding ranked according to CHD8 peak significance (Top 250 Bound, Rank 251–1000 Bound, Rank 1001–2000 Bound). Full models for certain datasets were chosen as they exhibited similar signal as the individual timepoint or brain region datasets.

TABLE S1 Log fold change expression data for all analyzed genes in each RNA-seq dataset according to a *p* < 0.05 or an FDR < 0.1 statistical cutoff.

TABLE S2 Normalized enrichment scores for all analyzed GSEA gene ontology and pathway terms for each RNA-seq dataset.

TABLE S3 Log_2_(observed/expected) enrichment scores for all analyzed goseq gene ontology terms according to different statistical thresholds (*p*-value < 0.05, FDR < 0.1, FDR < 0.05) for each RNA-seq dataset.

TABLE S4 Significance peak percentile values for all analyzed genes in each ChIP-seq dataset. 0 indicates no significant peak identified near the target gene.

TABLE S5 Normalized enrichment scores for analysis of overlap in ranked genes between RNA-seq and ChIP-seq datasets.
