# Supplementary figures and images for: Common CHD8 Genomic Targets Contrast With Model-Specific Transcriptional Impacts of CHD8 Haploinsufficiency
Source: Front Mol Neurosci. 2019 Jan 14;11:481. doi: 10.3389/fnmol.2018.00481 (PMC6339895; doi:10.3389/fnmol.2018.00481)

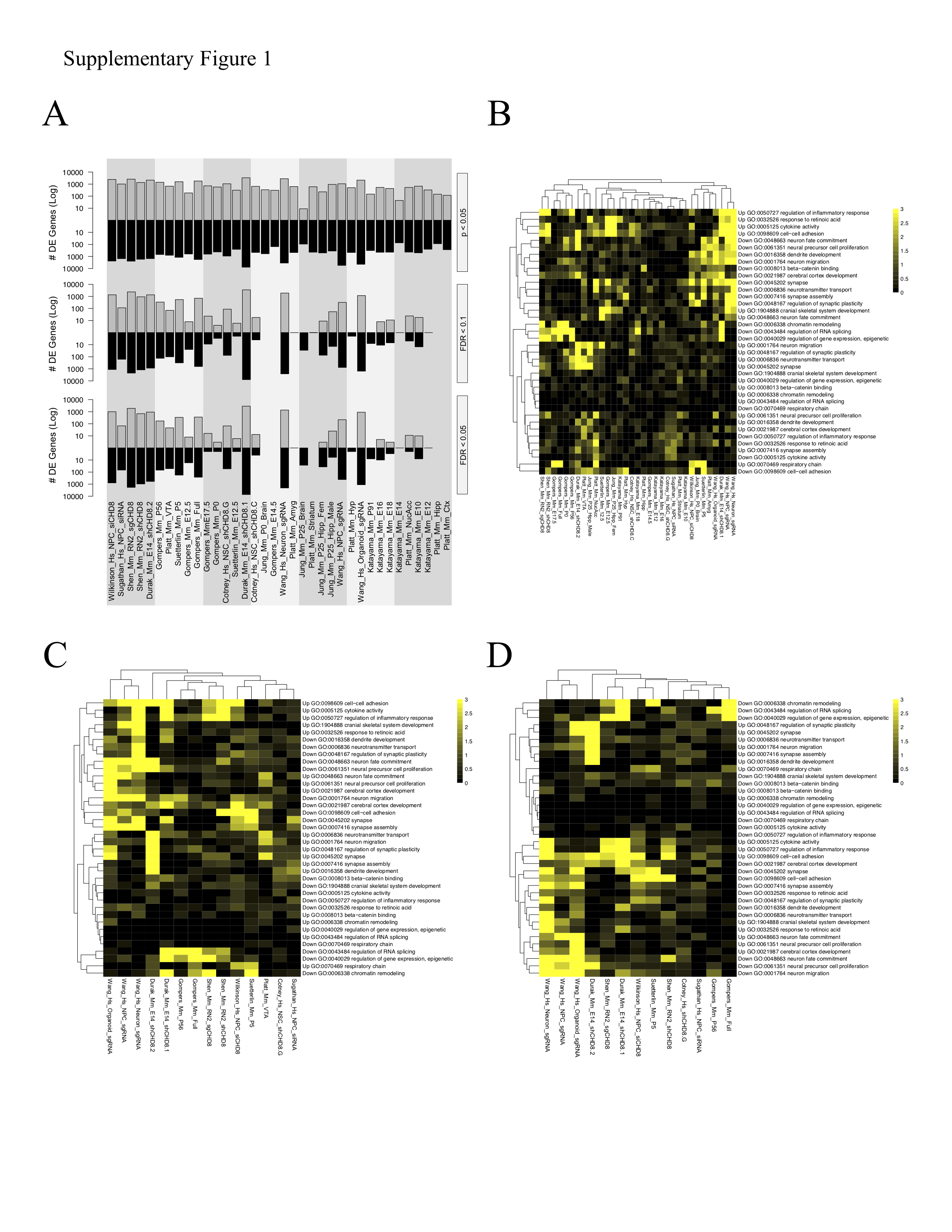

Supplement: FIGURE S1 — Consistency in RNA-seq results with goseq analysis across different statistical thresholds. (A) Bar plots showing log-scaled differentially expressed gene numbers for uncorrected p-value < 0.05 (top), FDR < 0.1 (middle), and FDR < 0.05 (bottom) thresholds with downregulated gene counts in black bars and upregulated gene counts in gray bars. (B–D) Heatmaps showing enrichment of gene regulation, neuronal function, and neurodevelopmental gene ontology terms when analyzing genes meeting a significance cutoff of p < 0.05 (B), FDR < 0.1 (C), and FDR < 0.05 (D) using the goseq statistical package. Included datasets are plotted on the x-axis. Significant terms are plotted on the y-axis for downregulated gene sets and upregulated gene sets separately, as indicated with “Up” and “Down,” at the beginning of each listed ontology term. The legend indicates log2(observed/expected) enrichment. Data were hierarchically clustered according to dataset similarity and term enrichment as indicated by dendrograms on the x- and y-axis. Ontology terms were selected to match terms for gene set enrichment analysis in Figure 1. Hs, human; Mm, mouse. [file Image_1.JPEG]

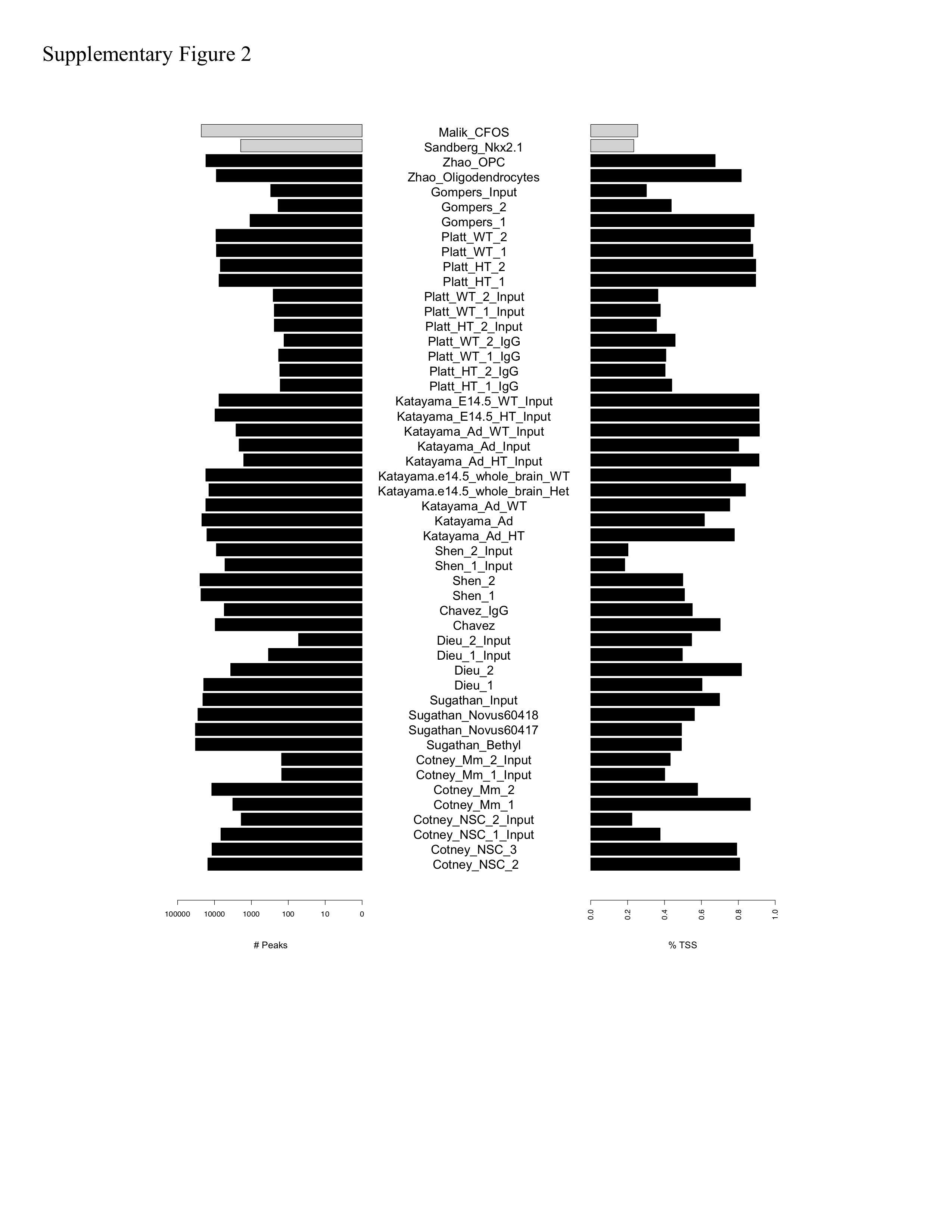

Supplement: FIGURE S2 — Significant peak number and preferential promoter binding by CHD8 (or control proteins) for all ChIP-seq datasets. Horizontal bar plots show the number of peaks meeting a MACS2 significance cutoff of p < 0.00001 (Left) and percentage of significant called peaks overlapping with the transcription start site of the nearest gene (Right). Control cFos (Malik) and Nkx2.1 (Sandberg) datasets are indicated with gray bars. [file Image_2.JPEG]

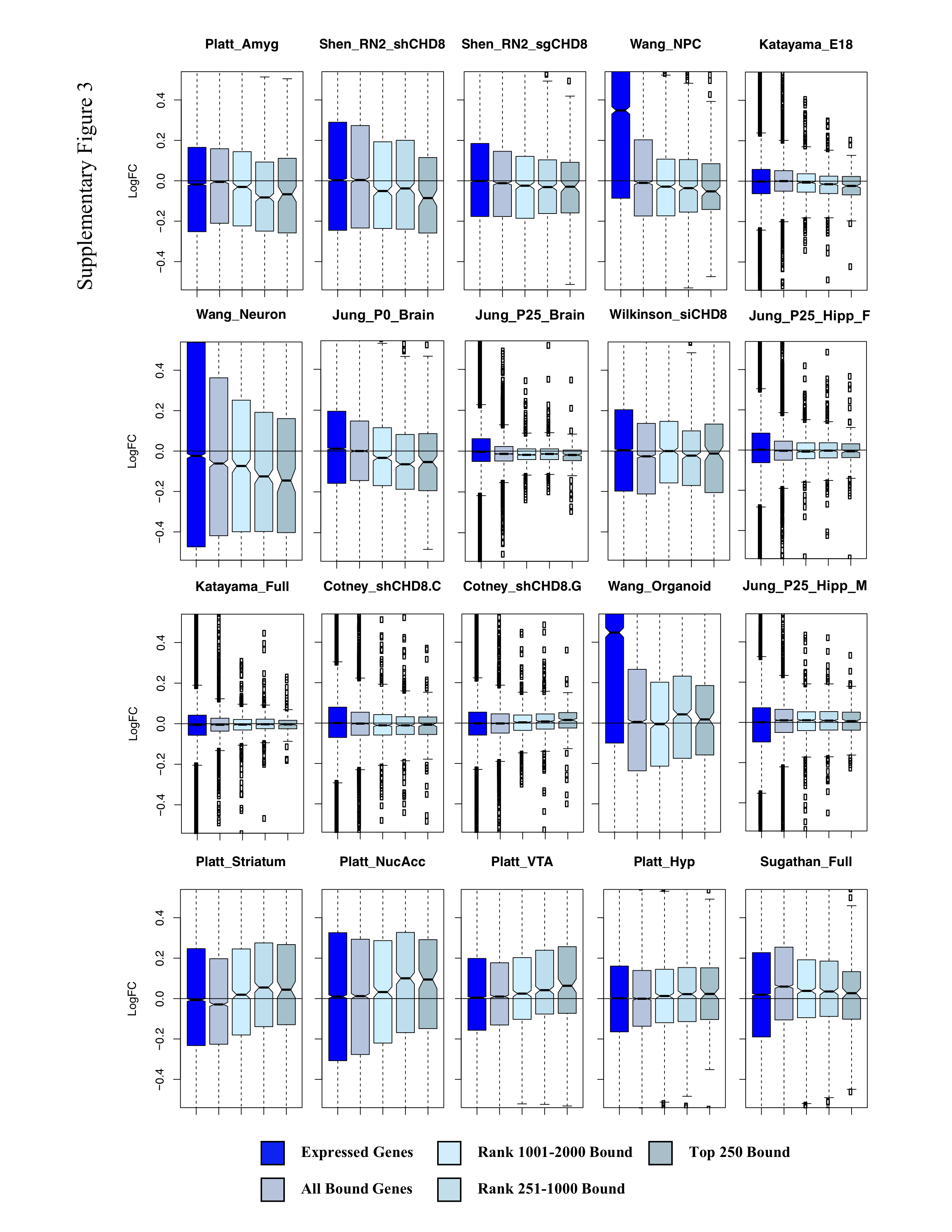

Supplement: FIGURE S3 — Remaining log fold change plots from the CHD8 binding by differential gene expression comparison analysis. All datasets were analyzed using the Platt et al. (2017) Chd8 ChIP-seq dataset. Datasets shown are from the CHD8 RNA-seq analysis and are loosely organized based on overlap between downregulated genes, no clear trend, or upregulated genes from top to bottom, which sometimes spanned multiple rows. Each plot shows log fold change on the y-axis and CHD8 binding affinity bin on the x-axis for each dataset as indicated by name above each plot. CHD8 binding affinity bins: all genes meeting at least 0.1 count per million sequencing coverage (Expressed Genes), any genes having CHD8 binding (All Bound Genes), and all genes having binding ranked according to CHD8 peak significance (Top 250 Bound, Rank 251–1000 Bound, Rank 1001–2000 Bound). Full models for certain datasets were chosen as they exhibited similar signal as the individual timepoint or brain region datasets. [file Image_3.JPEG]

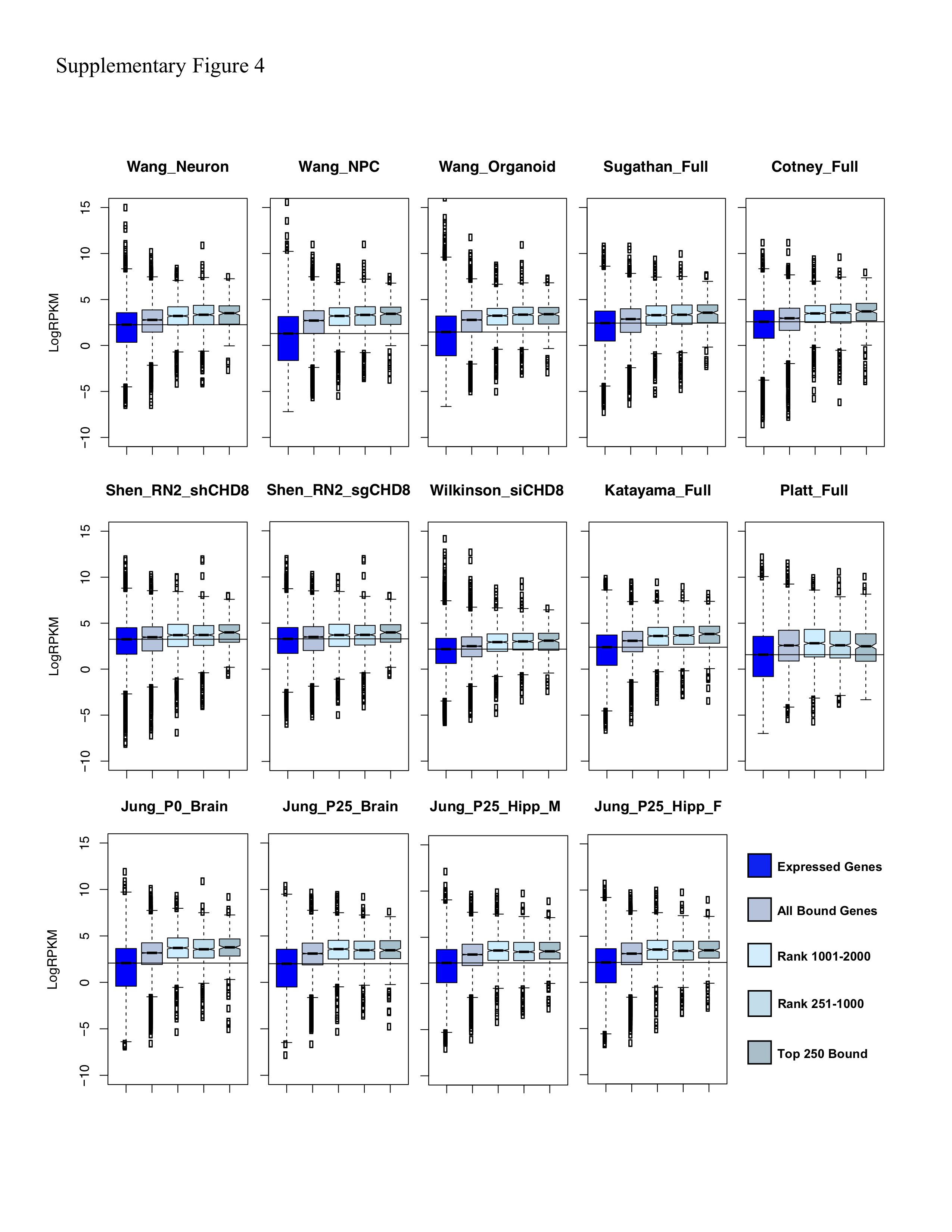

Supplement: FIGURE S4 — Remaining RPKM plots from the CHD8 binding by differential gene expression comparison analysis. All datasets were analyzed using the Platt et al. (2017) Chd8 ChIP-seq dataset. Datasets shown are from the CHD8 RNA-seq analysis. Each plot shows normalized log2RPKM on the y-axis and CHD8 binding affinity bin on the x-axis for each dataset as indicated by name above each plot. CHD8 binding affinity bins: all genes meeting at least 0.1 count per million sequencing coverage (Expressed Genes), any genes having CHD8 binding (All Bound Genes), and all genes having binding ranked according to CHD8 peak significance (Top 250 Bound, Rank 251–1000 Bound, Rank 1001–2000 Bound). Full models for certain datasets were chosen as they exhibited similar signal as the individual timepoint or brain region datasets. [file Image_4.JPEG]
